# Iodine-based dual-energy CT predicts early neurological decline from cerebral edema after large hemispheric infarction

**DOI:** 10.21203/rs.3.rs-3508427/v1

**Published:** 2023-11-10

**Authors:** William Denney Zimmerman, Melissa Pergakis, Ghasan Ahmad, Nicholas A Morris, Jamie Podell, Wan-Tsu Chang, Melissa Motta, Hegang Chen, Gaurav Jindal, Uttam Bodanapally, J. Marc Simard, Neeraj Badjatia, Gunjan Y Parikh

**Affiliations:** University of Maryland School of Medicine; University of Maryland School of Medicine; Hackensack Meridian Jersey Shore University Medical Center; University of Maryland School of Medicine; University of Maryland School of Medicine; University of Maryland School of Medicine; University of Maryland School of Medicine; University of Maryland School of Medicine; University of Maryland Medical Center; R Adams Cowley Shock Trauma Center; University of Maryland School of Medicine; University of Maryland School of Medicine; University of Maryland School of Medicine

## Abstract

**Background & Purpose:**

Ischemia affecting two thirds of the MCA territory predicts development of malignant cerebral edema. However, early infarcts are hard to diagnose on conventional head CT. We hypothesize that high-energy (190keV) virtual monochromatic images (VMI) from dual-energy CT (DECT) imaging enables earlier detection of secondary injury from malignant cerebral edema (MCE).

**Methods:**

Consecutive LHI patients with NIHSS ≥ 15 and DECT within 10 hours of reperfusion from May 2020 to March 2022 were included. We excluded patients with parenchymal hematoma-type 2 transformation. Retrospective analysis of clinical and novel variables included VMI Alberta Stroke Program Early CT Score (ASPECTS), total iodine content, and VMI infarct volume. Primary outcome was early neurological decline (END). Secondary outcomes included hemorrhagic transformation, decompressive craniectomy (DC), and medical treatment of MCE. Fisher’s exact test and Wilcoxon test were used for univariate analysis. Logistic regression was used to develop prediction models for categorical outcomes.

**Results:**

Eighty-four LHI patients with a median age of 67.5 [IQR 57,78] years and NIHSS 22 [IQR 18,25] were included. Twenty-nine patients had END. VMI ASPECTS, total iodine content, and VMI infarct volume were associated with END. VMI ASPECTS, VMI infarct volume, and total iodine content were predictors of END after adjusting for age, sex, initial NIHSS, and tPA administration, with a AUROC of 0.691 [0.572,0.810], 0.877 [0.800, 0.954], and 0.845 [0.750, 0.940]. By including all three predictors, the model achieved AUROC of 0.903 [0.84,0.97] and was cross validated by leave one out method with AUROC of 0.827.

**Conclusion:**

DECT with high-energy VMI and iodine quantification is superior to conventional CT ASPECTS and is a novel predictor for early neurological decline due to malignant cerebral edema after large hemispheric infarction.

## Introduction

Malignant cerebral edema (MCE) after large hemispheric infarction (LHI) leads to high levels of morbidity and mortality. Previous studies have investigated multiple variables linked to the development of malignant cerebral edema (MCE), especially early neurological decline (END) and life-threatening cerebral herniation after large hemispheric infarction. These clinical variables include the initial National Institute of Health stroke scale (NIHSS), the Glasgow coma scale (GCS), tissue-plasminogen activator (tPA) administration, serum biomarkers, and early CT and MRI imaging analyses.^1–4^ Initial computed tomography perfusion (CTP) ratio of cerebral blood volume (CBV) to cerebral spinal fluid (CSF) was shown to have a sensitivity and specificity greater than 95% for predicting a malignant infarct.^5^ However, in this small study, the primary outcome of malignant infarct included a decreased level of consciousness defined only as an increase in NIHSS ≥ 1. A prospective study of MRI diffusion-weighted imaging (DWI) infarct volume > 82 mL had a sensitivity of 98%, but low specificity for predicting malignant infarction.^6^ Using automated segmentation and a neural-based algorithm, it was shown that the initial CSF volume to tissue ratio and the progressive decline in CSF volume were predictors of cerebral edema formation.^3,4^ Nonetheless, routine identification of LHI patients at risk of clinical worsening and even death due to progressive malignant cerebral edema remains problematic.

The pathophysiology of cerebral edema after ischemic stroke is a complex, multifaceted cascade of events that includes cytotoxic, ionic, and vasogenic edema, all of which contribute to an increase in brain swelling. As ischemia progresses, the development of basal lamina damage occurs, resulting in an increase in microvascular permeability, which allows influx of proteinaceous fluid into the cerebral tissues.^7–11^ As edema progresses, oncotic cell death occurs and endothelial cell dysfunction results in extravasation of blood components, leading to hemorrhagic transformation (HT) and potentiation of further insult.^7,10,11^ Therefore, identifying individuals at risk of END due to MCE is a primary goal in initial triage and resuscitation of stroke patients with LHI. An estimated 50% of LHI patients experience END due to MCE in the first 2–3 days after admission, which is associated with higher risk of death, poorer functional outcomes and longer hospital stays.^12–15^ As the indications for acute mechanical thrombectomy for AIS with established large infarct continue to expand, the precedence of determining whom may worsen clinically, benefit from initial care at a comprehensive stroke center, or warrant acute neurosurgical intervention continues to elevate.^16,17^ Unfortunately, during the first 48 hours of LHI resuscitation, risk stratification generally depends on poorly-predictive data such as the neurological exam, midline shift, and clinical judgement. Advanced imaging techniques may address this practice gap by improving the rapid triage of these severely ill AIS patients.

The clinical utility of DECT in stroke has increased over the past decade, especially in the determination of an end stage of cerebral edema after AIS—hemorrhagic transformation. Through enhanced reconstructions from DECT, high-energy (190 keV) VMI allows the suppression of iodine attenuation, which has a similar pixel appearance as acute blood products on CT brain imaging.^18^ This CT technology also reduces beam hardening artifact, improves contrast resolution, and decreases image noise.^19^ Several studies have demonstrated high sensitivity for discriminating hemorrhagic transformation from iodine extravasation after mechanical thrombectomy (MT) utilizing dual-energy technology.^18,20,21^ VMI also has the ability to more accurately estimate the ischemic core compared to conventional non-contrast CT (NCCT) imaging.^22^ The likely explanation for this enhanced performance is VMI’s ability to highlight brain water content (BWC) and hypoattenuation, which on a fundamental level radiographically represents the early cascade of cytotoxic, ionic, and vasogenic edema.

Despite the improved visual delineation of cerebral edema and ability to distinguish between hemorrhagic transformation and iodinated contrast extravasation after AIS and MT, limited investigations exist quantifying iodine contrast extravasation on DECT as an imaging biomarker of blood brain barrier (BBB) disruption. To fulfill an unmet need for advanced techniques to rapidly quantify cerebral edema in LHI patients, routinely available imaging biomarkers must be established and may help identify those most appropriate for intensive neuromonitoring and early treatment in a comprehensive stroke center (CSC) and neurocritical care unit (NCCU). Like the concept of MRI hyperintense acute reperfusion marker (HARM) noted on FLAIR imaging due to gadolinium-based contrast agents crossing an early, disrupted BBB after AIS, we suspect that the leakage of iodinated contrast represents a CT-based equivalent, representative of cerebral edema development.^23^ As rapid MRI imaging procurement is not always clinically feasible for triage purposes, we postulate DECT’s ease of obtainment and performance may be the key to its widespread deployment. We hypothesize improved prediction of END due to MCE after LHI using high energy (190 keV) monochromatic images and quantification of iodinated contrast extravasation from DECT data.

## Methods

### Study Design and Participants

We completed a retrospective, observational study of a prospective, single institutional database (NCT0418947) of acute anterior circulation, large hemispheric infarction (LHI) patients who underwent intra-arterial (IA) mechanical thrombectomy (MT) and study-site standard post-reperfusion DECT within 10 hours of reperfusion. This study was approved by the University of Maryland School of Medicine Institutional Review Board and complies with ethical standards. For this type of retrospective study, informed consent is not required. LHI was defined as NIHSS ≥ 15 at the time of presentation of initial stroke syndrome. Exclusion criteria included patients who did not undergo standard post-IA therapy DECT imaging, significant post-MT clinical improvement defined as a 24-hour NIHSS < 12, and significant hemorrhagic transformation classified as parenchymal hematoma type 2 (PH-2). We followed the *Strengthening the reporting of observational studies in epidemiology* (STROBE) checklist in the design of this study.^24^

### Data Collection

Clinical data including age, sex, anterior stroke syndrome, tPA administration, stress glucose index, admission and 24-hour NIHSS and GCS, thrombolysis in cerebral infarction (TICI) score, and medical comorbidities including hypertension, previous cerebrovascular accident (CVA), congestive heart failure (CHF), renal dysfunction, diabetes mellitus, and stress glucose index were obtained through electronic medical review.^25–28^ Stress glucose index was determined by first calculating estimated average glucose from hemoglobin A1c (28.7 * A1c – 46.7), then calculating ratio of initial glucose measurement to estimated average glucose.^25,26^

### Definitions & Novel Variables

- **Iodine density**: software automated quantification in a ROI (region of interest) of brain parenchymal iodine extravasation measured in *mg/mL* on iodine images.
- **VMI infarct volume**: automated volumetric quantification of infarct volume (mL) on post-MT VMI CT.
- **Total iodine content**: total iodine extravasation (*mg*) post-MT quantified on iodine images in infarcted cerebral territory.

### Imaging Technical Analysis

We completed a retrospective analysis of both initial acute non-contrast, conventional CT (NCCT) and post-MT VMIs. DECT novel variables included quantified VMI CT ASPECTS and iodine extravasation variables including maximum iodine density in infarcted hemisphere, max iodine densities in corresponding ASPECTS locations, and calculated total iodine content in infarcted cerebral hemisphere. The study site utilized both Siemens and Philips DECT scanner technology for standard of care in management of AIS patients.

### Volume of Infarction

Using the Philips Intellispace Portal CT viewer application, we conducted infarct volumetric calculations of the high energy (190 keV) VMIs. Utilizing grey-level mapping, the 190 keV sequence was placed in a window width of W30:L30 or standard stroke viewing.^29^ A volume explorer application and “smart segmentation” tool allowed the use of a 3-D smart ROI to delineate the edges of infarcted tissue of the affected hemisphere. This border was adjusted along the infarct edge on each single axial slice to create a rendered lesion mapping and an automated infarct volume in milliliters (mL). To investigate the sensitivity of VMI infarct volume quantification in comparison to standard of care MRI DWI, two board certified neuroradiologists quantified final DWI infarct volume (mL) for first 21 patients with MRI imaging for comparison. Utilizing the manual lesion segmentation tool in Carestream Vue, PACS (Carestream Health Inc., Rochester, NY), the ROI delineating the infarct were manually segmented on individual slices from the DWI (b1000) sequence and the final infarct volume (mL) was obtained through automated summation.

### Iodine Extravasation Quantifications

For post image processing and iodine quantification, we implemented the modified brain hemorrhage application on the post-processing workstation (syngo.via, version VB10B; Siemens Healthcare, Forchheim, Germany) or the spectral CT viewer software (Philips Intellispace Portal; Philips Healthcare, Best, the Netherlands). For each DECT scan, the maximum iodine density in the affected hemisphere was determined using a free-hand ROI tool with automated calculation of iodine density (mg/mL). The final maximum iodine density was calculated by an averaged value in three planes: axial, coronal, and sagittal in the affected hemisphere. Further iodine density extrapolation was conducted by isolating each region of the ASPECTS score (caudate, insular, internal capsule, lentiform, M1, M2, M3, M4, M5, and M6) and again utilizing a ROI tool, an iodine density averaged in 3 planes was determined (mg/mL) in each corresponding location.

To estimate the novel DECT variable of total affected hemisphere iodine content (mg), we completed a 2-step process. First, the mean of the iodine densities (mg/mL) of the ten ASPECTS regions was calculated. Secondly, the product of the VMI infarct volumes (mL) and the mean iodine densities of the affected hemisphere (mg/mL) was determined, and this value was defined as the affected hemisphere total iodine content (mg).

### Outcomes

The primary outcome was defined as the development of END, using a composite outcome variable of both clinical worsening (increase NIHSS ≥ 4 or decrease in GCS > 2) or malignant radiographical edema (midline shift ≥ 5mm at the level of the septum pellucidum). Secondary outcomes included hemorrhagic transformation, implementing the Heidelberg bleeding classification (HI-1: scattered small petechiae, no mass effect, HI-2: confluent petechiae, no mass effect, or PH-1: hematoma within infarcted tissue, occupying < 30%, no mass effect), need for decompressive craniectomy (DC), and medical treatment of MCE with hyperosmolar therapy, sedation, or hyperventilation.^30^

### Statistical Analysis

Statistical analyses were performed by SAS version 9.4 (Cary, NC, USA). Fisher’s exact test and Wilcoxon Rank Sum test were used for univariate analysis. For multivariate analysis, both known clinical predictors of MCE defined by previous literature and variables of significance during the univariate analysis were fitted into the model. The summary statistics of the area under the receiver operating characteristic curve (AUROC) were calculated to measure prediction performance and the final model was cross validated by leave one out procedure. Linear regression modeling was used to relate MRI DWI infarct volumes to VMI infarct volumes to determine sensitivity of the VMI infarct volume calculation.

### Data Availability

Anonymized data not published within this article will be made available by request from any qualified investigator.

## Results

**Figure 1:**
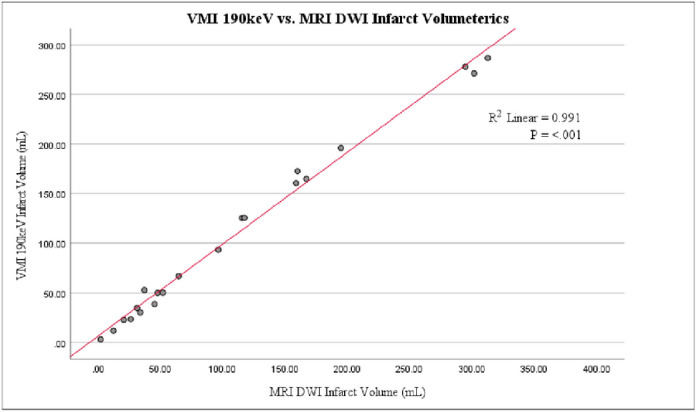
Regression line of VMI 190keV CT infarct volume vs. gold-standard MRI DWI infract volume of first 21 patients in cohort who underwent MRI imaging. (R^2^ = 0.991, p<0.001).

From May 2020 until March 2022, eighty-four LHI patients with median age of 67.5 [IQR 57,78] years and median initial NIHSS 22 [IQR 18,25] met inclusion criteria (Table 1). Forty-four (52.4%) patients were female, and majority of patients were either White (37 patients, 44%) or Black (45 patients, 53.6%). The two groups were well matched in relation to medical comorbidities other than those with HTN, who were less likely to have END (p=0.037) No differences were noted in volume of iodinated contrast administration (p=0.183), patients with congestive heart failure (CHF) (p=0.166), baseline serum creatinine (p=0.157) or GFR (p=0.509) in relation to contrast clearance. Linear regression of VMI infarct volume vs. MRI DWI infarct volume for first 21 patients who underwent MRI imaging demonstrated a coefficient of determination (R^2^) of 0.991, p<0.001 (Figure 1). No statistically significant difference was noted in 90-day post-discharge mRS (modified Rankin Score) (p=0.433) or when dichotomized to favorable (mRS ≤ 3) or unfavorable (mRS 4–5) outcome (p=0.594).

### Early Neurological Decline

**Figure 2:**
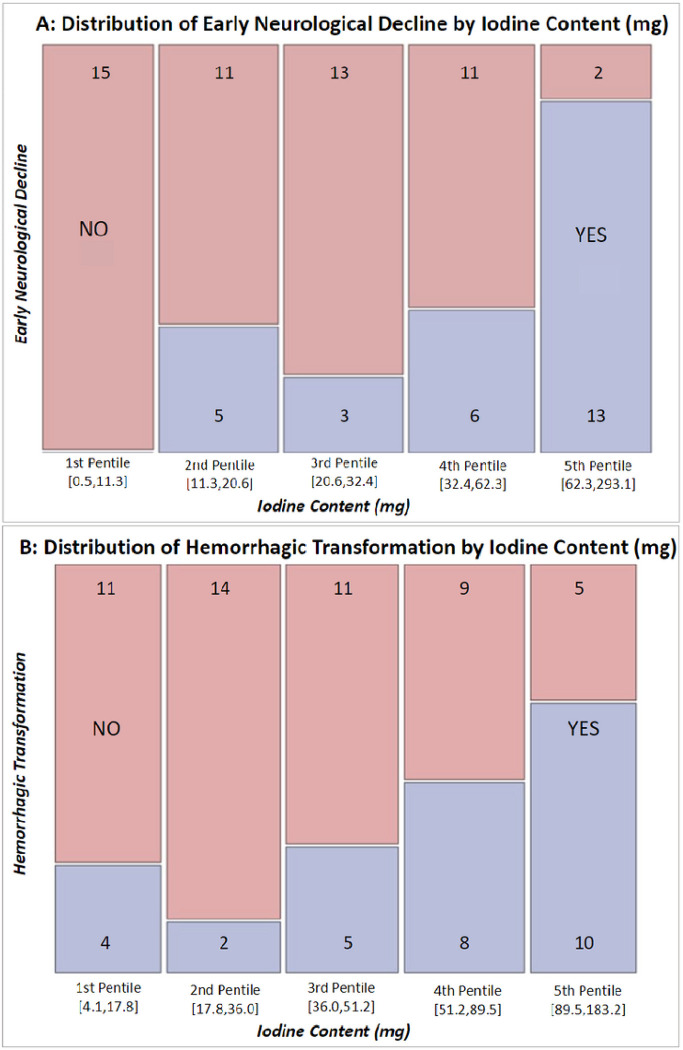
2A: Distribution of early neurological decline by iodine content (mg) in pentiles. 86.7% (13/15 patients) in the 5th pentile (Iodine content > 62.3 mg) met the primary endpoint of early neurological decline. 2B: Distribution of hemorrhagic transformation (HT) by iodine content (mg) in pentiles, demonstrating increased risk of HT with increasing iodine content.

**Figure 3:**
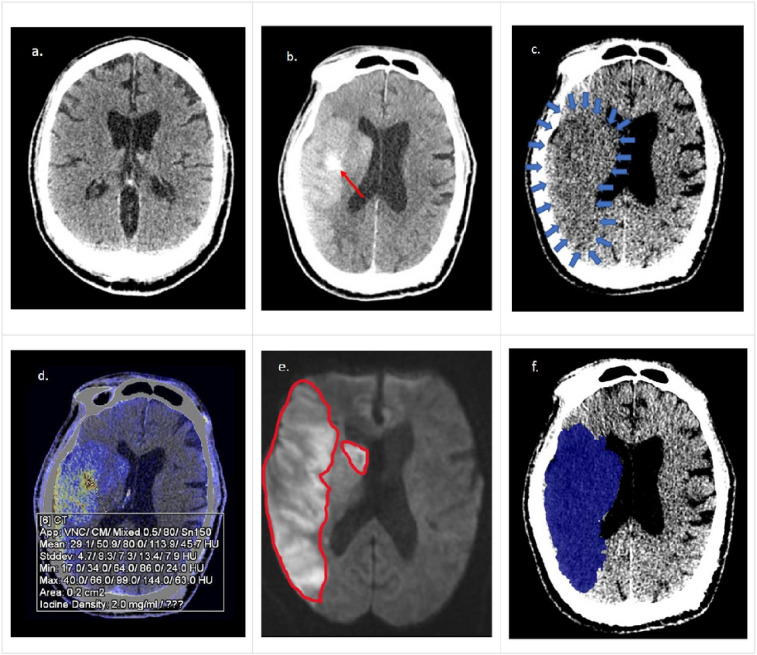
66-year-old male with past medical history of CHF & HTN, initial NIHSS 19 (R MCA syndrome), initial GCS 15, LKW to reperfusion (TICI 2c) 6 hours, 19 minutes, with GCS decline to 10 at 24 hours, received medical treatment for cerebral edema. 3a: Initial conventional NCCT (window width (WW): window length (WL), 70:30) with ASPECTS 10. 3b: Post-MT NCCT (WW:WL,70:30) demonstrating significant iodine contrast staining (red arrow) in R MCA ischemic tissue obscuring clear infarct margins. 3c: VMI 190 keV CT (WW:WL,30:30), noted delineation of hypodense cerebral tissue (blue arrows) with extraction of iodine contrast staining, no HT noted (LKW to DECT: 12 hours, 17 minutes). 3d: Modified brain hemorrhage application on the syngo-via workstation (Siemens) with an axial ROI with automated quantification of iodine density (2.0 mg/mL). 3e: MRI DWI (b1000) sequence with territory of ischemia (red line), with total MRI infarct volume quantification of 294.8 mL. 3f: Philips Intellispace Portal CT viewer application with volumetric quantification (blue shaded region) through axial segmentation and automated summation, total VMT infarct volume 278.0 mL.

A total of 29 patients (34.5%) met the primary endpoint of END, with 8 of the 29 (27.6%) patients having NIHSS increase ≥ 4, 21 (72.4%) patients developing MLS ≥ 5mm, and 22 (75.9%) patients having GCS decline > 2. Mean time from last known normal to meeting the primary endpoint was 41 hours and 49 minutes, with the END occurring on average on hospital day 2.03. In a univariate analysis, NIHSS at 24hrs., GCS at 24 and 48 hrs., post-MT ASPECTS on NCCT and VMI, delta ASPECTS between NCCT and VMI, and VMI infarct volume were all associated with END, with p-values noted in Table 1. The median VMI infarct was 159.9 mL in the END group vs. 62.4 mL in those not in the END group, p<0.001. Novel iodine related variables associated with END that were statistically significant include iodine densities (mg/mL) in the insular, internal capsule, M1, M4, M5, M6 locations (Table 2). Maximum iodine density for the affected hemisphere trended towards but did not reach significance (p=0.069). The median total iodine content for the END group was 62.3 mg vs. 21 mg, p<0.001 (Figure 2). In a linear regression comparing total iodine content and infarct volume, a positive correlation was found, R^2^ = 0.674, p <0.001 (Figure 3).

### Secondary Outcomes

In an analysis of the three secondary outcomes, 34 patients (40.1%) developed HT, 11 patients (13.1%) received medical treatment for MCE, and 7 patients (8.3%) underwent DC. Only one demographic variable was associated with HT—stress glucose ratio (p=0.034). Medical treatment for MCE was associated with race (p=0.032), younger age (p=0.003), and higher baseline creatinine (p=0.012). DC was associated with younger age (p=0.0002), higher baseline creatinine (p=0.0007), and lower GCS at 24 and 48 hours (p=0.02 and 0.014). With relation to novel variables, lower VMI ASPECTS was associated with all 3 secondary outcomes. Higher total iodine content was associated with HT (59.57 mg in HT group vs. 35.53 mg in non-HT group, p<0.05) and need for DC (65.3 mg in DC group vs. 37.9 mg in non-DC group, p=0.005). Increased VMI infarct volume was associated with medical treatment for MCE (163.6 mL medical treatment group vs. 99.1 mL non-treatment group, p=0.012) and DC (179 mL in DC group vs. 101.1 mL in non-DC group, p=0.013), but not HT. Iodine density in the deep locations of the ASPECTS including caudate, insular, internal capsule, and lentiform all were significantly higher in the HT group (p<0.05), but only the ID in the cortical locations of M1 (p=0.04) and M5 (p=0.013) were associated with DC.

### Multivariate Analysis

**Figure 4:**
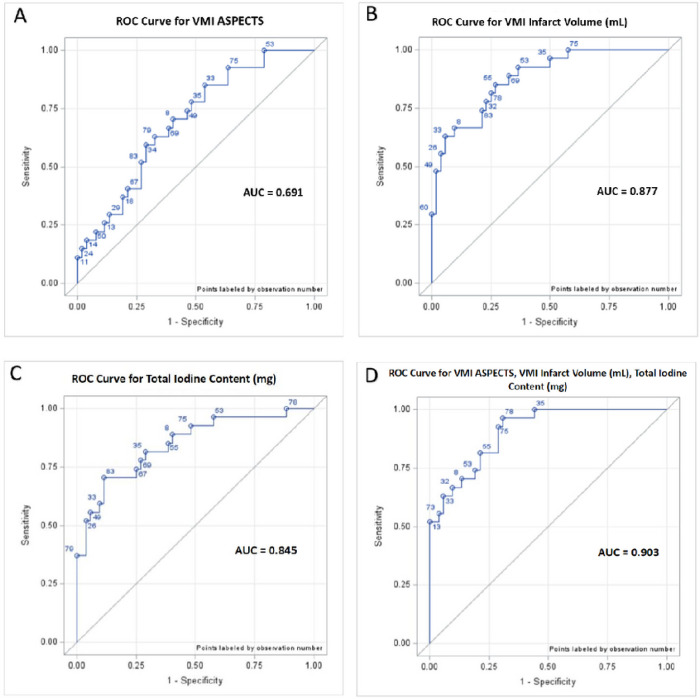
Multivariate regression analysis on prediction of neurological decline, standard variables for each model adjusted by age, sex, admission NIHSS, and tPA administration. 4A: Logistic regression model including variable: VMI ASPECTS (AUROC 0.691). 4B: Logistic regression model including variable: VMI infarct volume (mL) (AUROC 0.877). 4C: Logistic regression model including variable: total iodine content (AUROC 0.845). 4D: Final logistic regression model including variables: VMI ASPECTS, VMI infarct volume, and total iodine content (AUROC 0.903), with Leave 1 Out Cross Validation (LOOCV) (AUROC 0.827).

In a logistic regression analysis, VMI ASPECTS, VMI infarct volume, and total iodine content were predictors of END after adjusting for age, sex, initial NIHSS, and tPA administration, with an AUROC of 0.691 [0.57,0.81], 0.877 [0.80, 0.95], and 0.845 [0.75, 0.94] respectively (Figure 4A–C). In a final model, after adjusting for age, sex, initial NIHSS, and tPA administration, VMI ASPECTS, VMI infarct volume, and VMI iodine content, produced an AUROC of 0.903 [0.84,0.97] in predicting END, and was cross validated by leave one out procedure (AUROC of 0.827) (Figure 4D).

## Discussion

We investigated several novel DECT biomarkers using VMIs and iodine related variables to aid in the prediction of END after LHI. We used a composite score of END, including clinical decline and radiographical worsening, to capture the cascade of effects of malignant cerebral edema. In this at-risk population of LHI patients who underwent MT and post-reperfusion DECT imaging, we demonstrated that iodine contrast extravasation quantification, specifically total iodine content, and VMI infarct volume both were strong predictors of END. DECT in comparison to conventional CT, therefore better reflects total brain water content, BBB disruption, and the secondary injury that occurs through cytotoxic, ionic, and vasogenic edema.

The volume of ischemic injury can be rapidly measured after reperfusion without the need for MRI, as all VMIs from DECT data were obtained within 10 hours after MT. In comparison to gold-standard MRI DWI infarct volumes, VMIs delineate early ischemic changes equally well and outperforms conventional NCCT.^31^ An additional benefit of DECT is the rapid quantification of iodinated contrast. As infarct volume increased in this study, so did the quantity of iodinated contrast extravasation. This likely represents the degree of BBB disruption with a large contribution from reperfusion injury after MT secondary to hyperemia, continued cerebral metabolic depression and worsened paracellular permeability.^32^

Quantification of iodine contrast extravasation on DECT, specifically as a marker of BBB disruption, showed a strong association with secondary outcomes and appears to be a useful radiographic biomarker. Several studies have demonstrated the utility of DECT to differentiate acute blood products and iodinated contrast extravasation post-MT. Early differentiation is key, given most cases of post-thrombolytics or post-MT reperfusion hemorrhagic transformation occurs in the first 24 hours after intervention.^33,34^ In our cohort, larger infarct burden demonstrated by ASPECTS on both conventional NCCT and VMIs was associated with HT, with iodine leakage in the deep structures also associated with HT. Commonly, HT after AIS can be asymptomatic; however, it has been shown to worsen 3 month outcomes measured by mRS, suggesting another potential pharmacological target aimed at avoidance of endothelial dysfunction, oxidative stress, leukocyte infiltration, and vascular activation.^10,11,32^ Medical treatment for MCE was infrequent in this cohort, despite clinical and radiographical evidence of MCE. Medical treatment, unlike HT, was more associated with the total infarct volume of affected tissue as compared to the leakage of contrast only to deep structures. Surgical decompression occurred in less than 10% of our cohort, but similarly to medical treatment, occurred in younger patients with worsened baseline renal function both likely due to clinical decision making and limitations in ICP monitoring. Quantification of both tissue injury by infarct volume and BBB disruption through total iodine content were associated with the need for DC. All patients who underwent DC did so in the first 4 days after onset of stroke symptoms. Early identification of LHI patients who require DC and appropriate triage to a comprehensive stroke center is paramount.

The retrospective review design of this study is the primary limitation. Despite excluding END due to fever, sedation, or seizure, confounding by unmeasured variables remains possible. Routine use of DECT imaging on initial evaluation, pre-MT, did not occur until midway through this study, therefore we were unable to compare initial VMIs to post-MT imaging, a future direction of this work. Contrast clearance post-MT may be impacted by heart failure or renal disfunction. The administration of contrast during MT is protocolized with no difference between the patients who developed END and those who did not. Also, our cohort is small, although our goal was proof of clinical utility and prospective evaluation is necessary moving forward.

Future investigations must be directed towards utilization of the high energy (190 keV) VMI derived from DECT as the initial imaging modality in acute stroke investigation which is now standard of care at our center. Given its success at determining the infarct core compared to MRI, we anticipate its greatest value lies in its use as a clinical triage tool when evaluating risk of early neurological decline^22^ Automated quantification of iodinated contrast extravasation and infarct volumetrics will aid in the rapid utility of the DECT in AIS triage. The ease of obtainment, readily available technology, and shortened time outside of the ICU make DECT imaging a promising acute neuroimaging modality after LHI.^35^

## Conclusion

VMIs and iodine quantification from DECT is superior to conventional CT ASPECTS for demonstration of cerebral infarction burden and is a novel predictor for early neurological decline due to malignant cerebral edema after large hemispheric infarction.

## Figures and Tables

**TABLE 1:** Baseline Characteristics

	All patients (n = 84)	END^1^ (n = 29)	No END (n = 55)	p-value

Age	67.5 [57.0,78.0]	64.0 [48.5,80.0]	71.0 [59.0,78.0]	0.185

Sex				
Female	44 (52.4%)	16 (55.2%)	28 (50.9%)	0.710

Race				
White	37 (44.0%)	9 (31.3%)	28 (50.9%)	
Black	45 (53.6%)	18 (62.1%)	27 (49.1%)	0.107
Hispanic	1 (0.6%)	1 (3.4%)	0 (0.0%)	
Asian	1 (0.6%)	1 (3.4%)	0 (0.0%)	

Medical History				
Hypertension	61 (72.6%)	17 (58.6%)	44 (80.0%)	0.037
History of CVA^2^/TIA^3^	17 (20.2%)	4 (13.8%)	13 (23.6%)	0.286
CHF^4^ (EF<40%)	24 (38.6%)	7 (24.1%)	17 (30.9%)	0.514
Diabetes Mellitus	28 (33.3%)	9 (31.0%)	19 (34.5%)	0.746
Tobacco Use	34 (40.5%)	10 (34.5%)	24 (43.6%)	0.416

Serum Creatinine	1.14 [0.76,1.2]	0.99 [0.77,1.26]	0.9 [0.74,1.16]	0.157

GFR^5^	73.5 [56.5,94.5]	73.0 [47.0,97.5]	75.0 [58.0,93.0]	0.509

Iodinated Contrast (cc)	85.0 [60.0,130.0]	100.0 [67.5,140.0]	80.0 [60.0,130.0]	0.183

Stress Glucose Ratio	1.08 [0.91,1.2]	1.15 [1.0,1.28]	1.05 [0.86,1.14]	0.143

tPA^6^ Administration	31 (36.9%)	12 (41.4%)	19 (34.6%)	0.537

Stroke Syndrome				
R MCA^7^	27 (32.4%)	13 (44.8%)	14 (25.5%)	
L MCA	39 (46.4%)	12 (41.4%)	27 (49.1%)	0.254
R ICA^8^/MCA	3 (3.6%)	1 (3.5%)	2 (3.6%)	
L ICA/MCA	15 (17.9%)	3 (10.3%)	12 (21.8%)	

TICI^9^ score ≥ 2B	75 (89.3%)	25 (86.2%)	50 (90.9%)	0.508

Admission NIHSS^10^	22 [18,25]	22 [19,24]	22 [18, 26]	0.983

24-hour NIHSS	20 [16,23]	22 [19,25]	19 [15,22]	0.008

Admission GCS^11^	10 [8.5,12.5]	11 [8.5,13.5]	10.0 [8,12]	0.243

24-hour GCS	10 [8,11]	9 [8,10]	10 [8,13]	0.015

48-hour GCS	10 [8,12]	9 [7,10]	11.0 [9,13]	<.0001

Blood Pressure				
Initial SBP^12^	160 [139.5,179.5]	166.0 [140.0,183.0]	159.0 [139.0,177.0]	0.474
Initial MAP^13^	110 [100.5,125.5]	116 [101.0,127.5]	107 [100,122]	0.359
Post MT SBP	147.5 [126,169]	162 [133,169]	143 [127,169]	0.101
Post MT MAP	102.5 [87,115]	106 [94,120]	100 [86,113]	0.147

ASPECTS^14^ Initial NCCT^15^	9 [7,9.5]	9 [7,9.5]	9 [7,10]	0.882

ASPECTS Post-MT^16^ NCCT	6 [5,7.5]	6 [4,7]	7 [6,8]	0.009

ASPECTS VMI^17^ 190keV CT	4 [2,6]	3 [1.5,5]	5 [3,6]	0.003

Delta ASPECTS (Initial NCCT vs. VMI CT	4 [3,5]	5 [3,7]	3 [2,5]	0.007

Mean ASPECTS Iodine Density (mg/mL)	0.32 [0.22,0.46]	0.42 [0.25,0.53]	0.3 [0.21,0.38]	0.148

VMI CT Infarct Volume (mL)	91.0 [50.9,144.2]	159.9 [99.5,233.9]	62.4 [38.1,99.3]	<.0001

Iodine Content (mg)	27.1 [12.6,53.3]	62.3 [23.9,128.1]	21.0 [8.8,32.1]	<.0001

Time Variables (hh:mm)				
LKW^18^ to Reperfusion	6:40 [4:59,12:18]	7:25 [4:42,14:28]	6:29 [4:59,12:14]	0.787
LKW to VMI CT	12:10 [9:37,17:10]	12:17 [8:59,18:58]	12:07 [9:50,15:54]	0.647
Reperfusion to VMI CT	4:55 [3:54,5:48]	4:23 [2:58,6:03]	5:17 [4:21,5:41]	0.293
LKW to END	---	35:29 [24:07,57:51]	---	---
VMI CT to END	---	16:43 [6:13,39:08]	---	---

90-day mRS^19^ 0–6	4.0 [2.0,6.0]	4.0 [2.0,6.0]	5.0 [2.0,6.0]	0.433
Death (mRS 6) Dichotomized mRS	30 (36.1%)	14 (48.3%)	16 (29.6%)	
Favorable: mRS ≤ 3	27 (32.5%)	10 (34.5%)	17 (30.9%)	0.128
Unfavorable: mRS 4–5	26 (31.3%)	5 (17.2%)	21 (38.2%)	

1 END, Early neurological decline,

2 CVA, cerebrovascular accident,

3 TIA, transient ischemic attack,

4 CHF, congestive heart failure,

5 GFR, glomerular filtration rate,

6 tPA, tissue plasminogen activator,

7 MCA, middle cerebral artery,

8 ICA, internal carotid artery,

9 TICI, thrombolysis in cerebral infarction score,

10 NIHSS, National Institute of Health Stroke Scale,

11 GCS, Glascow coma scale,

12 SBP, systolic blood pressure,

13 MAP, mean arterial blood pressure,

14 ASPECTS, Alberta stroke program early CT score,

15 NCCT, non-contrasted computed tomography,

16 MT, mechanical thrombectomy,

17 VMI, virtual monochromatic images,

18 LKW, last known well,

19 mRS, Modified Rankin Scale

**TABLE 2:** Univariate Analysis of Novel Iodine Densities and Iodine Content of Infarcted Hemisphere

	All patients (n = 79)	END^1^ (n = 27)	No END (n = 52)	p-value
Caudate ID^2^ (mg/mL)	0.26 [0.19,0.39]	0.22 [0.18,0.48]	0.27 [0.19,0.35]	0.599
Insular ID (mg/mL)	0.31 [0.2,0.54]	0.44 [0.29,0.71]	0.30 [0.2,0.5]	0.037
Internal capsule ID (mg/mL)	0.3 [0.11,0.6]	0.4 [0.18,0.7]	0.21 [0.1,0.45]	0.043
Lentiform ID (mg/mL)	0.37 [0.2,0.76]	0.4 [0.21,0.8]	0.31 [0.2,0.5]	0.196
M1^3^ ID (mg/mL)	0.23 [0.2,0.76]	0.37 [0.2,0.43]	0.2 [0.1,0.3]	0.006
M2^4^ ID (mg/mL)	0.37 [0.2,0.5]	0.41 [0.3,0.55]	0.3 [0.2,0.5]	0.181
M3^5^ ID (mg/mL)	0.26 [0.13,0.4]	0.3 [0.13,0.44]	0.25 [0.12,0.37]	0.330
M4^6^ ID (mg/mL)	0.3 [0.1,0.41]	0.37 [0.2,0.58]	0.2 [0.1,0.37]	0.010
M5^7^ ID (mg/mL)	0.29 [0.19,0.45]	0.43 [0.21,0.6]	0.26 [0.15,0.35]	0.010
M6^8^ ID (mg/mL)	0.25 [0.15,0.33]	0.3 [0.2,0.5]	0.22 [0.11,0.32]	0.001
Maximum Hemispheric Iodine Density (ID) (mg/mL)	0.78 [0.6,1.3]	0.97 [0.60,1.53]	0.76 [0.49,1.08]	0.069
Mean ASPECTS^9^ Iodine Density (mg/mL)	0.32 [0.22,0.46]	0.42 [0.25,0.53]	0.3 [0.21,0.38]	0.148
Iodine Content (mg) of infarcted hemisphere	27.1 [12.6,53.3]	62.3 [23.9,128.1]	21.0 [8.8,32.1]	<.0001

1 END, early neurological decline,

2 ID, iodine density,

3 M1: anterior middle cerebral artery (MCA) cortex, corresponding to frontal operculum;

4 M2: MCA cortex lateral to the insular ribbon,

5 M3: posterior MCA cortex,

6 M4: anterior MCA territory superior to M1,

7 M5: lateral MCA territory superior to M2;

8 M6: posterior MCA territory superior to M3,

9 ASPECTS, Alberta Stroke Program early CT score
